# X-Ray Crystal Structure and Solution Properties of [Au(III)(H_-2_-Gly-Gly-L-His)]Cl.H_2_O

**DOI:** 10.1155/MBD.1994.510

**Published:** 1994

**Authors:** Sabine L. Best, Tapan K. Chattopadhyay, Milos I. Djuran, Muhammed A. Mazid, Rex A. Palmer, Peter Sadler

**Affiliations:** 1 Department of Chemistry Birkbeck College University of London 29 Gordon Square London WC1 OPP United Kingdom; 2 Department of Crystallography Birkbeck College University of London Malet Street London WC1E 7HX United Kingdom


					X-RAY CRYSTAL STRUCTURE AND SOLUTION PROPERTIES OF
[Au(III)(H.2-Gly-Gly-L-His)]CI.H20
Sabine L. Best,Tapan K. Chattopadhyay,Milos I. Djuran,
Muhammed A. Mazid2, Rex A. Palmer2and and Peter Sadler
Department of Chemistry, Birkbeck College, University of London,
29 Gordon Square, London WC10PP, UK
2 Department of Crystallography, Birkbeck College, University of London,
Malet Street, London WC1E 7HX, UK
Gold(Ill) generated via myeloperoxidase is thought to play an important role in the side-effects of
anti-arthritic gold drugs [1, 2]. Au(lll)-modified peptides bound to MHC class II molecules may be
involved in delayed hypersensitivity reactions ('gold rashes') observed in patients undergoing
chrysothempy, but little is known about the binding of Au(lll) to peptides.
Only one crystal structure of a Au(lll) peptide has previously been described [3].
For our studies, we used glycylglycyI-L-histidine (GGH), a model for the N-terminal copper binding
site of serum albumin. The reaction of GGH with NaAuCI4 at low pH (1.5-2) is slow (T1/2 ca. 6 h,
10 mM, 310 K) and involves one intermediate. The structure of the final product [Au(III)(H.2-Gly-
Gly-L-His)]CI.H20 has been determined by X-my crystallography. It is square-planar with Au(lll)
coordinated to the terminal NH2, two deprotonated peptide NH's and His NS. The structure of this
complex in solution and its behaviour at pH 7 will be discussed.
[1]
[2]
[3]
B. Beverly and D. Couri, Fed. Proc. 46:854 (1987)
D. Schuhmann, M. Kubicka-Mumnyi, J. Mirtschewa, J. G0nther, P. Kind, and E.
Gleichmann, J. Immunol. 145:2132-2139 (1990)
M. Wienken, B. Lippert, E. Zangrando, and L. Randaccio, Inorg. Chem. 31:1983-1985 (1992)
We thank the Wellcome Trust, Association for International Cancer Reseamh, MRC, SERC, Royal
Society and ULIRS for their support.
510

				

